# Synthesis, Computational Studies and Anticonvulsant Activity of Novel Benzothiazole Coupled Sulfonamide Derivatives

**Published:** 2019

**Authors:** Sukhbir L. Khokra, Kanika Arora, Shah A. Khan, Pawan Kaushik, Reetu Saini, Asif Husain

**Affiliations:** a *Institute of Pharmaceutical Sciences, Kurukshetra University, Kurukshetra-136119, Haryana, India. *; b *Department of Pharmacy, Oman Medical College, Muscat, Sultanate of Oman. *; c *Department of Pharmaceutical Chemistry, School of Pharmaceutical Education and Research (SPER), Jamia Hamdard, New Delhi, 110062, India.*

**Keywords:** Anticonvulsant, Benzenesulfonamide, Benzothiazole, Computational analysis, MES

## Abstract

We report herein the synthesis of ¾ substituted benzene sulfonamides linked via phenyl ring to a benzothiazole moiety. The title compounds in the two series namely *N*-(4-(benzothiazole-2-yl) phenyl) 4- substituted benzene sulfonamides and *N*-(4-(benzothiazole-2-yl) phenyl) 3- substituted benzene sulfonamides were synthesized by condensing 2-(3/4-aminophenyl) benzothiazole with various substituted sulfonyl chlorides. The synthesized compounds were subjected to neurotoxicity screening, computational studies, and evaluation of their anticonvulsant potential. Amongst all the synthesized compounds, compound **9** emerged as the most potent anticonvulsant agent in maximal electroshock (MES) model (standard: phenytoin) in mice and showed three hydrogen bond interactions with the nicotinic acetylcholine ion gated receptors (PDB ID: 2BG9). Interestingly, compound **13** showed five hydrogen bond interactions with the target protein and thus excellent binding affinity upon computational analysis but was found to be neurotoxic.

## Introduction

Epilepsy is one of the most common, non-communicable neurological disorder of central nervous system (CNS) affecting approximately 1-2% of total world’s population. It is characterized by the sudden recurrent seizures that occur primarily due to a sudden increase in the electrical activity in the brain (1). There are over twenty drugs available to treat this destructive condition; however, only six or seven out of them are used in clinical practice. With the existing anti-convulsant drug therapy approximately 1/4^th^ of epileptic patients are inadequately controlled and moreover their use is also associated with serious side effects (2, 3). Therefore, there is a need to develop safer, potent, and less toxic anti-convulsant drug candidates. 

**Figure 1:**
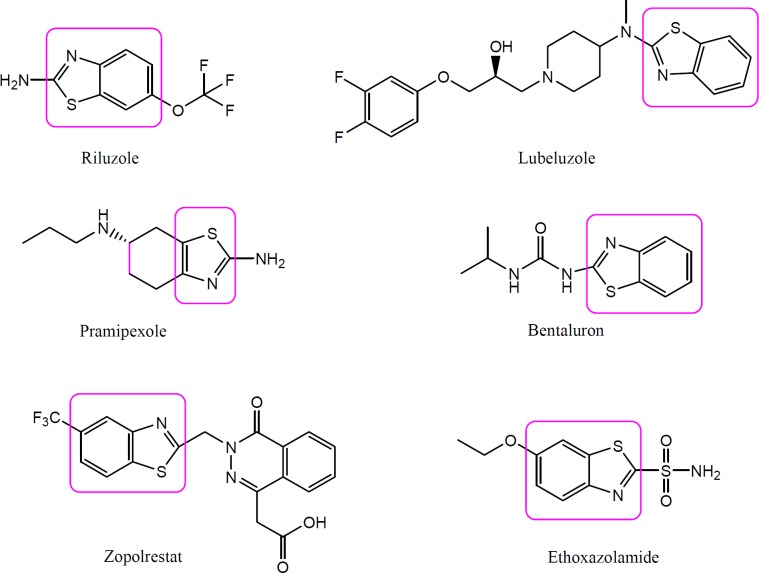
Chemical structures of few drugs containing benzothiazole nucleus

Benzothiazole is a bicyclic organosulfur heterocyclic compound which is weakly basic in nature (4). Benzothiazole is a biologically active moiety present in several natural products in which benzene is fused with a five membered ring thiazole at position 4 and 5. Natural products containing benzothiazole nucleus are mainly found in *Asparagus racemosus*, cocoa, mango, roasted peanuts and filberts, cassava roots, soyabean milk, stored casein, sterilized concentrated milk, thymol oil, *etc* (5). Benzothiazoles and their derivatives have attracted a great deal of interest recently due to their wide range of useful biological activities such as anticancer, antimicrobial, anti-tubercular, anti-HIV, cardiovascular, local anaesthetic, anti-inflammatory, anti-convulsant, and anti-diabetic (6-10). In addition, benzothiazole pharmacophore is known to be present in various clinically useful drugs such as Pramipexole, Lubeluzole, Zopolrestat, Ethoxzolamide, Bentaluron, and Riluzole, etc., indicating that it possesses diverse biological actions (Figure 1). Riluzole, a benzothiazole containing moiety, is having phenytoin like spectrum of anticonvulsant activity. Beside this, a number of benzothiazole derivatives as well as derivatives of riluzole have been reported to exhibit anticonvulsant activity in preliminary experimental studies (10, 11). Hence, compounds containing benzothiazole nucleus appears to be potential and promising candidates for the development of anti-convulsant therapy. 

**Figure 2:**
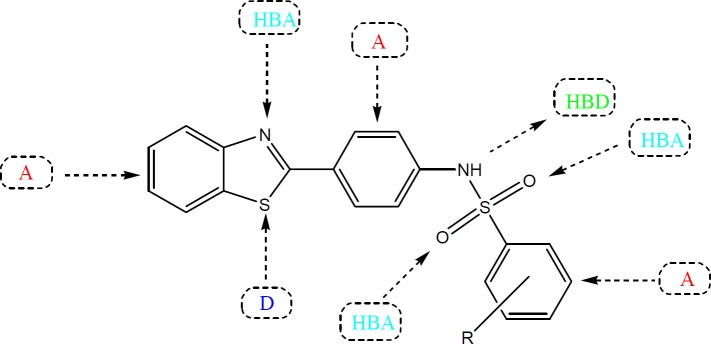
General chemical structure of title compounds showing essential pharmacophoric features of anticonvulsant agents (A: hydrophobic aryl ring, D: Electron donor atom, HBA; Hydrogn bond acceptor; HBD: Hydrogen bond donor).

Several studies have been carried out in the past to improve the therapeutic potential and utility of benzothiazole containing compounds by either direct incorporation of various functional groups in benzothiazole nucleus or fusion with heterocyclic rings that resulted in significant alteration of their pharmacokinetic/pharmacodynamic properties (12). The substituted benzothiazole compounds are comparatively easy to prepare and possess characteristic pharmacological features due to the presence of an inbuilt biologically active unit. Some of other advantages of substitution include relative stability, enhanced lipid solubility and ease of metabolism *in-vivo* by routine biochemical reactions (13). In lieu of these observations, we decided to prepare some new benzothiazole derivatives bearing a sulfonamide group with an aim to investigate their anti-convulsant activity. The title compounds possess the essential pharmacophoric features of an ideal anticonvulsant agent which are required for binding at the receptor site. In general, the benzothiazole coupled sulfonamide derivatives contain aromatic ring (A) as lipophilic domain that could assist in crossing the blood brain barrier, an electron donor system (D) in form of S and N atoms and a hydrogen bonding domain [Hydrogn bond acceptor (HBA)/Hydrogen bond donor (HBD)] in form of a NH-(O=S=O) group in their chemical structure for the possible interactions with the target proteins (Figure 2) (14).

Computer assisted drug designing (CADD) is an emerging field of computational medicinal chemistry which has become an important technique for the chemists to discover, design and optimize biologically active lead compounds with desired structure and properties with a putative use as drugs (15, 16). Docking is a powerful computational tool in structural molecular biology that is used to carry out virtual screening on large libraries of compounds in short time. Molecular docking helps in evaluation of the structural hypotheses that involve inhibition of the target by the ligands (17). The main aim of the ligand protein docking is to predict the predominant binding mode (s) of a ligand with a protein of known three dimensional structures. Molecular docking is playing an important role in drug design and discovery, including structure activity relationship (SAR) studies, lead optimization, potential lead identification by virtual screening, chemical mechanism studies, *etc* (18). The most important and interesting aspect of drug design is to predict the binding of a small molecule to a target macromolecule. Taking into consideration about all these findings, it was felt worthwhile to carry out the synthesis of some novel benzothiazole sulfonamide derivatives and perform molecular docking studies to identify the compounds possessing better anti-convulsant activities than the existing drug molecules. 

## Experimental

*Materials and Methods*

Chemicals of high purity were obtained from E. Merck India Ltd, CDH, S D Fine- Chem Limited, India and Qualigens Fine Chemicals, India. Melting points were taken on slides in an electrical apparatus (Lab India visual melting range apparatus) and are uncorrected. IR spectra were recorded on Jasco FT-IR spectrophotometer and *v*_max_values are given in cm^-^^1^. ^1^H and ^13^C NMR spectra were recorded in CDCl_3_ and DMSO-d_6_ on a Bruker Nuclear Magnetic Resonance (NMR) spectrometer at 400 MHz and 100 MHz respectively using tetramethylsilane (TMS) as an internal reference. Chemical shifts are expressed in delta (δ). Mass spectra were recorded on a Jeol SX- 102/DA-6000 (Tokyo, Japan) spectrometer. 

*Synthesis of substituted benzothiazole derivatives*

*Synthesis of 2-(3/4-amino phenyl) benzothiazole derivatives*

Accurately weighed quantities of molecular iodine (0.008 mM) and 3/4-amino benzoic acid (0.0084 mM) were placed in a mortar. Then, 2-aminothiophenol (0.008 mM) was added drop wise to the mortar with trituration. The progress of reaction was monitored with the help of TLC (toluene: ethyl acetate: formic acid, 5:4:1). Trituration was continued for approximately 10 min *i.e.* till the completion of reaction. The crude solid product was recrystallized in 70% methanol. 

*Synthesis of different substituted benzene sulfonyl chlorides*

Benzene or its substituted derivatives (0.148 M) was placed in a two necked flask with a dropping funnel and a reflex condenser. Chlorosulfonic acid (0.77 M) was taken in a dropping funnel with a calcium chloride guard tube attached to it. The chlorosulfonic acid was added in small portions with stirring. The resulting mixture was further heated on a water bath for one hour. The oily mixture was cooled and poured in a thin stream into a beaker containing crushed ice. The flask was rinsed with ice water and the rinsing was added to the beaker. 

The mixture was stirred to obtain the sulfonyl chloride derivative which was filtered off. The product was washed with cold water and dried (19).

*Synthesis of N-(4-(benzothiazole-2-yl) phenyl) ¾ substituted benzene sulfonamides*

2-(3/4-amino phenyl) benzothiazole derivatives (0.01 M) was added to the mixture of pyridine (4 mL) and acetic anhydride (20 mL). After the addition of benzene sulfonyl chlorides (0.01 M) to the above mixture, it was heated on a water bath for 2 h. 

The reaction mixture was poured onto 30 mL of water and the solid product so obtained was filtered and recrystallized from 80% ethanol (20).

*N-(4-(benzothiazole-2-yl) phenyl) benzenesulfonamide (* ***1*** *)*

mp. 236-238 °C; yield 56%; R_f_ 0.64; IR (*v*_max_, KBr, cm^-1^): 3333 (N-H_str_), 3090 (-SO_2_NH_str_), 1625 (C=N), 1468, 1356 (C-N_str_), 1142; ^1^H NMR (CDCl_3_): δ 4.34 (s, 1H, NH), 7.22-7.89 (m, 13H, Ar-H); ^13^C NMR (CDCl_3_): δ 115.1-148.5 (18C, Ar), 168.6 (1C, -C=N); (MS: *m/z *366(M^+^; C_19_H_14_N_2_O_2_S_2_), 368(M^+^+2).

*N-(4-(benzothiazole-2-yl) phenyl)-4-chlorobenzene sulfonamide* ***(2)***

mp. 296-299 °C; yield 48%; R_f_ 0.76, IR (*v*_max_, KBr, cm^-1^): 3352(N-H_str_), 3115 (-SO_2_NH_str_), 1654(C=N), 1453,1370(C-N_str_), 1145, 694; ^1^H NMR (CDCl_3_): δ 4.23 (s, 1H, NH), 7.15-7.89 (m, 12H, Ar-H); ^13^C NMR (CDCl_3_): δ 115.3-151.2 (18C, Ar), 169.3 (1C, -C=N); MS: *m/z *400(M^+^; C_19_H_13_ClN_2_O_2_S_2_), 402(M^+^+2).

*N-(4-(benzothiazole-2-yl) phenyl)-4-bromobenzenesulfonamide* ***(3)***

mp. 220-223 °C; yield 53%; R_f_ 0.79; IR (*v*_max_, KBr, cm^-1^): 3333(N-H_str_), 3108 (-SO_2_NH_str_), 1612(C=N), 1474, 1389(C-N_str_), 1142, 687; ^1^H NMR (DMSO-d_6_): δ 4.70 (s, 1H, NH), 7.36-7.97 (m, 12H, Ar-H);^ 13^C NMR (DMSO-d_6_): δ 115.3-148.7 (18C, Ar), 168.6 (1C, -C=N); MS: *m/z *444(M^+^; C_19_H_13_BrN_2_O_2_S_2_), 446 (M^+^+2).

*N-(4-(benzothiazole-2-yl) phenyl)-4-fluorobenzenesulfonamide* ***(4)***

mp. 274-278 °C; yield 41%; R_f_ 0.73; IR (*v*_max_, KBr, cm^-1^): 3318(N-H_str_), 3119 (-SO_2_NH_str_), 1650(C=N), 1465,1345(C-N_str_), 1156; ^1^H NMR (DMSO-d_6_): δ 4.58 (s, 1H, NH), 7.16-7.78 (m, 12H, Ar-H);^ 13^C NMR (DMSO-d_6_): δ 115.3-148.8 (18C, Ar), 168.9 (1C, -C=N); MS: *m/z *384(M^+^; C_19_H_13_FN_2_O_2_S_2_), 386 (M^+^+2).

*N-(4-(benzothiazole-2-yl) phenyl)-4-methylbenzene sulfonamide* ***(5)***

mp. 225-227 °C; yield 67%; R_f_ 0.75; IR (*v*_max_, KBr, cm^-1^): 3392(N-H_str_),3084 (-SO_2_NH_str_), 1658(C=N), 1445, 1352(C-N_str_), 1163; ^1^H NMR (CDCl_3_): δ 2.50 (s, 3H, CH_3_), 4.11 (s, 1H, NH), 7.12-7.90 (m, 12H, Ar-H);^ 13^C NMR (CDCl_3_): δ 21.4 (1C, -CH_3_),114.9-147.4 (18C, Ar), 169.1 (1C, -C=N); MS: *m/z *380(M^+^; C_20_H_16_N_2_O_2_S_2_), 382(M^+^+2).

*N-(4-(benzothiazole-2-yl) phenyl)-4-nitrobenzene sulfonamide* ***(6)***

mp. 245-248 °C; yield 59%; R_f_ 0.81; IR (*v*_max_, KBr, cm^-1^): 3279(N-H_str_), 3088 (-SO_2_NH_str_), 1651(C=N), 1504, 1319(C-N_str_), 1149; ^1^H NMR (CDCl_3_): δ 4.19 (s, 1H, NH), 7.26-8.13 (m, 12H, Ar-H);^ 13^C NMR (CDCl_3_): δ 114.9-149.9 (18C, Ar), 170.1 (1C, -C=N); MS: *m/z *411 (M^+^; C_19_H_13_N_3_O_4_S_2_), 413(M^+^+2).

*N-(4-(benzothiazole-2-yl) phenyl)-4-bromo-3-methylbenzenesulfonamide* ***(7)***

mp. 264-267 °C; yield 39%; R_f_ 0.84; IR(*v*_max_, KBr, cm^-1^): 3418(N-H_str_), 3112 (-SO_2_NH_str_), 1595(C=N), 1445, 1352(C-N_str_), 1150, 635; ^1^H NMR (DMSO-d_6_): δ 2.67 (s, 3H, CH_3_), 4.39 (s, 1H, NH), 7.29-8.01 (m, 12H, Ar-H);^ 13^C NMR (DMSO-d_6_): δ 18.9 (1C, -CH_3_),116.2-149.1 (18C, Ar), 169.8 (1C, -C=N); MS: *m/z *458 (M^+^; C_20_H_15_BrN_2_O_2_S_2_), 460 (M^+^+2).

*N-(4-(benzothiazole-2-yl) phenyl)-3-benzenesulfonamide* ***(8)***

mp. 224-225 °C; yield 58%; R_f_ 0.69; IR (*v*_max_, KBr, cm^-1^): 3455(N-H_str_),3085 (-SO_2_NH_str_), 1620(C=N), 1470, 1350(C-N_str_), 1145; ^1^H NMR (DMSO-d_6_): δ 4.46 (s, 1H, NH), 7.20-7.83 (m, 13H, Ar-H);^ 13^C NMR (DMSO-d_6_): δ 115.6-148.1 (18C, Ar), 169.8 (1C, -C=N); MS: *m/z *366(M^+^; C_19_H_14_N_2_O_2_S_2_), 368(M^+^+2).

*N-(4-(benzothiazole-2-yl) phenyl)-3-chlorobenzenesulfonamide* ***(9)***

mp. 268-271°C; yield 49%; R_f_ 0.72; IR (*v*_max_, KBr, cm^-1^): 3375(N-H_str_),3102 (-SO_2_NH_str_), 1640(C=N), 1445, 1370(C-N_str_), 1155, 705; ^1^H NMR (CDCl_3_): δ 4.39 (s, 1H, NH), 7.28-7.87 (m, 12H, Ar-H);^ 13^C NMR (CDCl_3_): δ 115.5-149.9 (18C, Ar), 169.2 (1C, -C=N); MS: *m/z *400(M^+^; C_19_H_13_ClN_2_O_2_S_2_), 402(M^+^+2).

*N-(4-(benzothiazole-2-yl) phenyl)-3-bromobenzenesulfonamide* ***(10)***

mp. 211-214 °C; yield 48%; R_f_ 0.74; IR (*v*_max_, KBr, cm^-1^): 3425(N-H_str_),3068 (-SO_2_NH_str_), 1636(C=N), 1470, 1355(C-N_str_), 1145; ^1^H NMR (CDCl_3_): δ 4.51 (s, 1H, NH), 7.37-7.91 (m, 12H, Ar-H);^ 13^C NMR (CDCl_3_): δ 115.6-149.8 (18C, Ar), 169.2 (1C, -C=N); MS: *m/z *444(M^+^; C_19_H_13_BrN_2_O_2_S_2_), 446 (M^+^+2).

*N-(4-(benzothiazole-2-yl) phenyl)-3-fluorobenzene sulfonamide* ***(11)***

mp. 252-255 °C; yield 46%; R_f_ 0.69; IR (*v*_max_, KBr, cm^-1^): 3401(N-H_str_),3095 (-SO_2_NH_str_), 1650(C=N), 1465, 1365(C-N_str_), 1160; ^1^H NMR (CDCl_3_): δ 4.34 (s, 1H, NH), 7.21-7.78 (m, 12H, Ar-H);^ 13^C NMR (CDCl_3_): δ 115.6-149.9 (18C, Ar), 169.8 (1C, -C=N); MS: *m/z *384(M^+^; C_19_H_13_FN_2_O_2_S_2_), 386(M^+^+2).

*N-(4-(benzothiazole-2-yl) phenyl)-3-methylbenzenesulfonamide* ***(12)***

mp. 184-186 °C; yield 62%; R_f_ 0.78; IR (*v*_max_, KBr, cm^-1^): 3355(N-H_str_),3078 (-SO_2_NH_str_), 1650(C=N), 1445, 1345(C-N_str_), 1150; ^1^H NMR (CDCl_3_): δ 2.38 (s, 3H, CH_3_), 4.56 (s, 1H, NH), 7.15-7.77 (m, 12H, Ar-H);^ 13^C NMR (CDCl_3_): δ 19.8(1C, -CH_3_), 115.3-147.2 (18C, Ar), 168.9 (1C, -C=N); MS: *m/z *380(M^+^; C_20_H_16_N_2_O_2_S_2_), 382(M^+^+2).

*N-(4-(benzothiazole-2-yl) phenyl)-3-nitrobenzenesulfonamide* ***(13)***

mp. 232-235 °C; yield 55%; R_f_ 0.83; IR (*v*_max_, KBr, cm^-1^): 3410(N-H_str_), 3085 (-SO_2_NH_str_), 1640(C=N), 1460, 1365(C-N_str_), 1160; ^1^H NMR (CDCl_3_): d 4.19 (s, 1H, NH), 7.19-8.06 (m, 12H, Ar-H);^ 13^C NMR (CDCl_3_): d 115.9-150.1 (18C, Ar), 169.7 (1C, -C=N); MS: *m/z *411(M^+^; C_19_H_13_N_3_O_4_S_2_), 413(M^+^+2).

*N-(4-(benzothiazole-2-yl) phenyl)-4-bromo-3-methylbenzenesulfonamide* ***(14)***

mp. 251-254 °C; yield 42%; R_f_ 0.87; IR (*v*_max_, KBr, cm^-1^): 3378(N-H_str_),3092 (-SO_2_NH_str_), 1590(C=N), 1495, 1350(C-N_str_), 1150, 630; ^1^H NMR (CDCl_3_): d 2.51 (s, 3H, CH_3_), 4.32 (s, 1H, NH), 7.23-8.01 (m, 12H, Ar-H);^ 13^C NMR (CDCl_3_): d 18.1 (1C, -CH_3_),115.9-149.8 (18C, Ar), 169.6 (1C, -C=N); MS: *m/z *458(M^+^; C_20_H_15_BrN_2_O_2_S_2_), 460 (M^+^+2).

*Pharmacological assay*

*Animals*

Animal ethical clearance approval was obtained for the use of animals in the current study from Institutional Ethical Committee (IEC). Albino male mice weighing 25-30 gm were used in this study. A total of 5 animals were kept in each polypropylene cage under laboratory conditions with controlled environment of temperature 25 ± 2°C and 12 h light/dark cycle as per CPCSEA guidelines. The bedding of cages was made with dust free rice husk and animals were given free access to drinking water and fed *ad libitum* with standard laboratory rodent’s chow food. The animals were acclimatized with laboratory conditions for a period of one week. The animals were fasted overnight prior to the experiment.

*Drug*

Pentylenetetarazole (PTZ) – was used to induce convulsions. Phenytoin sodium and diazepam were used as standards in Maximal electroshock seizures (MES) and PTZ methods respectively. The solution of test compounds/drugs was prepared in 0.9 w/v% normal saline and then administered intraperitoneally (*i.p.*) in volumes of 0.1 mL/25 g of body weight.

**Table 1:** Effect of synthesized compounds on maximal electroshock (MES) in mice

**S. No.**	**Treatment (Comp no.)**	**Phases of convulsions: Mean ± SEM**				
		**Flexion**	**Extension**	**Clonus**	**Stupor**	**Recovery**
1	Control	5.2 ± 0.58	12.6 ± 0.68	6.4 ± 0.68	12.4 ± 0.51	186.2 ± 5.3
2	Standard	3.6 ± 0.68*	0*	1.6 ± 0.24*	1.6 ± 0.24*	171.8 ± 2.92
3	1	3.4 ± 0.51*	1.8 ± 0.37*	4.4 ± 0.75*	15.2 ± 1.07	145.2 ± 4.5*
4	2	2.8 ± 0.37*	1.6 ± 0.24*	4.8 ± 0.37*	16.6 ± 1.36	126.8 ± 208*
5	3	8.4 ± 0.51	1.8 ± 0.58*	5.0 ± 0.71	21 ± 2.24	131.4 ± 5.15*
6	4	3.2 ± 0.86*	1.6 ± 0.40*	4.2 ± 0.86*	18 ± 3.56	127.8 ± 4.55*
7	5	4.6 ± 0.51	2.4 ± 0.51*	4.4 ± 0.75*	18.4 ± 2.06	128.6 ± 6.04*
8	6	2.4 ± 0.51*	1.8 ± 0.37*	4.0 ± 0.71*	17.8 ± 1.86	135.6 ± 0.69*
9	7	2.2 ± 0.37*	2.4 ± 0.51*	4.8 ± 0.58	18 ± 2.07	133.8 ± 8.42*
10	8	2.2 ± 0.58	1.6 ± 0.40*	4.4 ± 0.51	19.8 ± 1.32	129.2 ± 3.67*
11	9	1.8 ± 0.37*	1.2 ± 0.20*	3.8 ± 0.8*	2.4 ± 2.83*	129 ± 5.32*
12	10	3.4 ± 0.40*	1.4 ± 0.20*	5.2 ± 0.86	19.4 ± 2.5	135.8 ± 11.2*
13	11	2.2 ± 0.58*	1.4 ± 0.40*	4.8 ± 1.16*	18 ± 2.07	120.4 ± 3.71*
14	12	2.4 ± 0.74*	1.6 ± 0.60*	4.0 ± 0.70*	17.8 ± 2.40	132.6 ± 3.01*
15	13	2.2 ± 0.73*	2.0 ± 0.77*	5.0 ± 0.86	17.6 ± 3.31	128.8 ± 6.09*
16	14	2.6 ± 0.4*	1.8 ± 0.58*	5.0 ± 1.14	15.8 ± 1.99	130.2 ± 3.65*

The data were analysed by one-way ANOVA followed by Dunnett’s test,

* *p *> 0.01 compared to control group.

**Table 2:** Percentage inhibition in extensor phase

**S. No.**	**Treatment (Compound No.)**	**% Inhibition in extensor phase (as compared to control group)**
1	Control	0
2	Standard	98.41
3	1	85.71
4	2	87.30
5	3	85.71
6	4	87.30
7	5	80.95
8	6	85.71
9	7	80.95
10	8	87.30
11	9	90.47
12	10	88.88
13	11	88.88
14	12	87.30
15	13	84.12
16	14	85.71

**Table 3:** Effect of synthesized compounds on PTZ test in mice

**S. No.**	**Treatment (Compound No)**	**Onset of convulsions**	**Onset of action (Mean ± SEM)**	**Quantal death**
1	Control	3.6 ± 0.51	10.8 ± 0.73	5/5
2	Standard	0*	0	0/5
3	1	3.8 ± 0.37	10.6 ± 1.21	5/5
4	2	3.6 ± 0.81	13.0 ± 1.58	5/5
5	3	3.2 ± 0.37	12.6 ± 0.93	5/5
6	4	3.0 ± 0.32	12.8 ± 0.86	5/5
7	5	3.2 ± 0.58	17.6 ± 2.44	5/5
8	6	3.0 ± 0.71	19.0 ± 2.97	5/5
9	7	3.0 ± 1.05	16.6 ± 1.63	5/5
10	8	1.8 ± 0.37*	16.6 ± 3.75	5/5
11	9	2.2 ± 0.49*	18.4 ± 1.63	5/5
12	10	2.4 ± 0.75*	13.4 ± 1.33	5/5
13	11	3.8 ± 0.49	14.6 ± 1.97	5/5
14	12	3.8 ± 0.58	13.6 ± 1.36	5/5
15	13	3.4 ± 1.03	14.6 ± 1.97	5/5
16	14	3.0 ± 0.77	15.0 ± 1.55	5/5

The data were analysed by one-way ANOVA followed by Dunnett’s test,

* *p *> 0.01 compared to control group.

**Table 4:** Compounds showing interactions between synthesized compounds and amino acids residues with different potency (PDB ID: 2BG9)

S. No.	Structure	Mol Dock Score	Docking Score	H-bonds	H-bonds distance (Å)	Interacting Residue	Interacting Molecule
**Standard**	EPS_1050 [A]	-116.65	-113.74	3	2.60, 3.16, 2.90	Arg 277, Arg 277, Gln 276	O of SO_2_-N, O of SO_2_-N, N of NH-O
**1**	H	-106.33	-107.38	2	2.85, 2.68	Arg 277, Arg 277	O of SO_2_-N, O of SO_2_-N
**2**	Cl	-110.31	-110.90	2	2.90, 3.00	Arg 277, Arg 277	O of SO_2_-N, O of SO_2_-N
**3**	Br	-111.83	-112.06	2	3.10, 2.96	Arg 277, Gln 276	O of SO_2_-N, N of thiazole-O
**4**	F	-107.09	-107.16	2	2.72, 3.32	Arg 277, Arg 277	O of SO_2_-N, O of SO_2_-N
**5**	CH_3_	-110.72	-111.97	2	2.60, 3.20	Arg 277, Arg 277	O of SO_2_-N, O of SO_2_-N
**6**	NO_2_	-114.67	-118.59	3	3.88, 2.42, 2.84	Arg 277, Arg 277, Arg 277	N of NO_2_-N, N of NO_2_-N, O of NO_2_-N
**7**	Br, CH_3_	-112.39	-113.68	2	2.81, 3.21	Glu 262, Glu 274	N of NH-O, N of thiazole-O
**8**	H	-104.49	-104.69	2	2.60, 3.21	Arg 277, Arg 277	O of SO_2_-N, O of SO_2_-N
**9**	Cl	-104.72	-109.47	3	3.21, 2.93, 2.92	Gln 276, Tyr 223, Tyr 223	O of SO_2_-N, O of SO_2_-N, O of SO_2_-O
**10**	Br	-103.75	106.22	3	3.37, 2.57, 3.18	Tyr 223, Tyr 223, Lys 269	N of NH-O, O of SO_2_-O, O of SO_2_-N
**11**	F	-113.84	-112.07	3	3.14, 3.24, 2.91	Arg 277, Glu 262, Arg 277	O of SO_2_-N, N of NH-O, O of SO_2_-N
**12**	CH_3_	-107.75	-106.90	1	2.92	Glu 262	N of thiazole-O
**13**	NO_2_	-115.37	-120.35	5	3.29, 3.25, 3.45, 2.59, 2.85	Arg 277, Arg 277, Ser 266, Arg 277, Arg 277	N of NO_2_-N, N of NO_2_-N, O of SO_2_-O, O of NO_2_-N, O of NO_2_-N
**14**	CH_3,_Br	-110.09	-110.29	1	3.50	Glu 274	O of SO_2_-O

**Figure 3:**
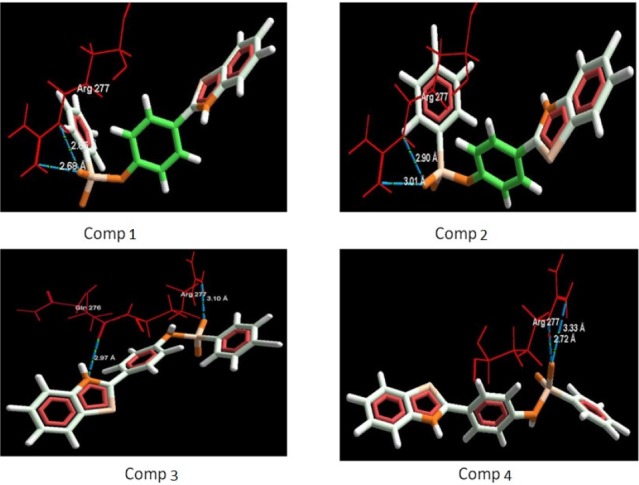
Binding mode of compounds (1-4) in to nicotinic acetylcholine ion gated receptors. Hydrogen bonds are shown with blue dotted lines

**Figure 4.**
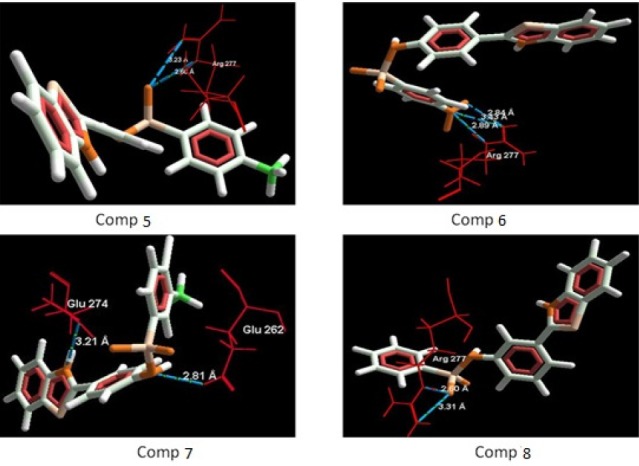
Binding mode of compounds (5-8) in to nicotinic acetylcholine ion gated receptors. Hydrogen bonds are shown with blue dotted lines.

**Figure 5:**
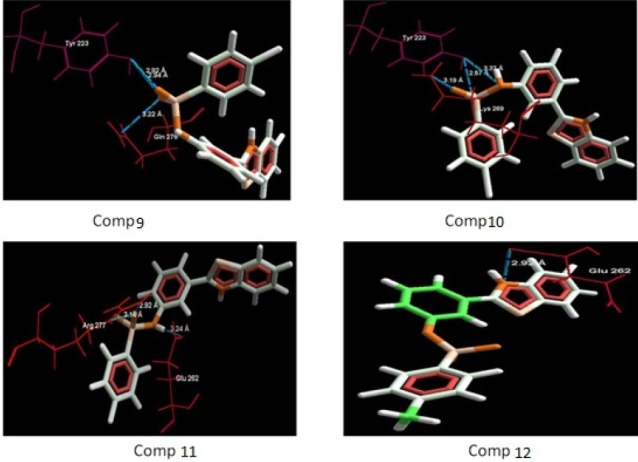
Binding mode of compounds (9-12) in to nicotinic acetylcholine ion gated receptors. Hydrogen bonds are shown with blue dotted lines

**Figure 6:**
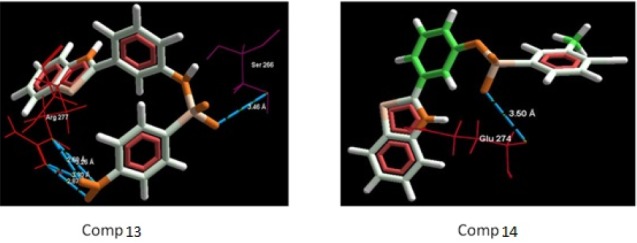
Binding mode of compounds (13-14) in to nicotinic acetylcholine ion gated receptors. Hydrogen bonds are shown with blue dotted lines

**Scheme 1:**
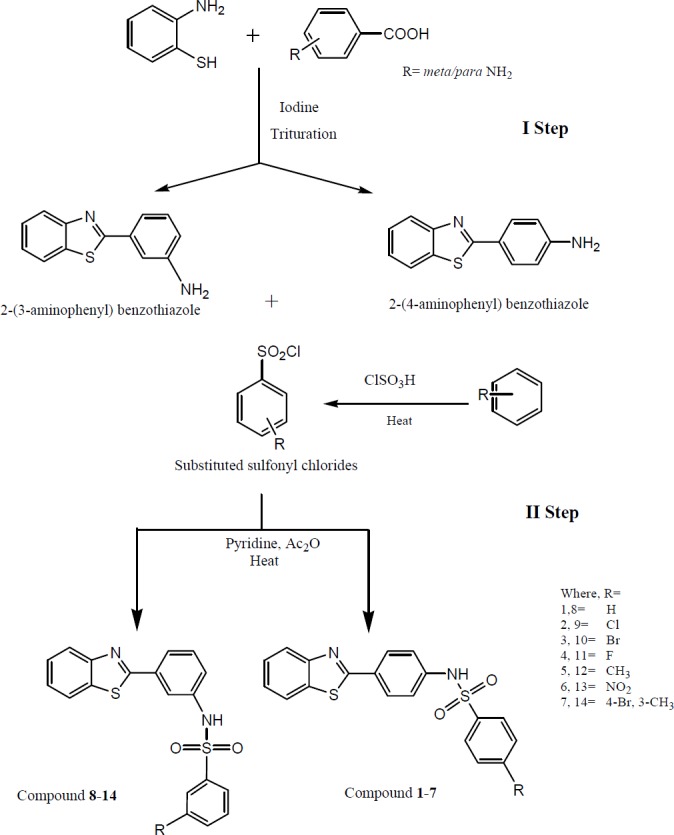
Synthesis of *N*-[(4-benzothiazole-2-yl) phenyl] ¾ substituted benzenesulfonamides

*Anti-convulsant activity*

*Maximal electroshock (MES) method*

In MES method, an electrical stimulus (12 mA, 50 Hz, 0.2 sec duration) was applied through ear-clip electrodes to induce tonic hind limb extension (THLE). The mice which showed extension of hind limb in preliminary screening were chosen for the current study. Sixteen groups (1-16) of five mice each pre-treated with normal saline: tween (10 mL/kg, as control), phenytoin (25 mg/kg as positive control) and test drugs (50 mg/kg), received the electroconvulsive shock 60 min later. The time of peak effect of phenytoin was previously established. After electrical stimulation, occurrence of THLE, duration and incidence of mortality were noted. Disappearance of the hind limb extensor tonic convulsion is used as positive criterion. Percentage of inhibition of seizures relative to controls is calculated as shown in Table 1. The abolition in time of tonic extensor phase of MES-convulsions was recorded (21)

*PTZ induced seizures method*

PTZ is a CNS stimulant that produces jerky type of clonic convulsions in rat and mice. The minimal *i.p.* dose of PTZ at which 99% of the animals induce hind limb tonic extension (HLTE) was selected from the literature data (22). PTZ at the dose of 60 mg/kg b.w. (minimal dose needed to induce convulsions) was injected *i.p.* to induce clonic-tonic convulsions in animals. The groups (3-16) of animals (n = 5) received test compounds (50 mg/kg b.w, *p.o*), the positive control group 2 received standard drug diazepam (1 mg/kg *i.p*) and normal control group 1 was given normal saline: tween (10 mL/kg, *p.o*). After 60 min of pre-treatment with test compounds/standard drug, PTZ (*i.p.*) was administered to all sixteen groups and mice were observed for 30 min to detect the occurrence of general clonus, HLTE, and mortality. The delay of onset was calculated in comparison with the control group. 

*Neurotoxicity screening*

The effect of synthesized compounds on neurotoxicity or minimal motor impairment in the mice was assessed by the rotarod test as per the standard method (23). Albino mice were trained to stay for at least one minute on an accelerating rotating rod of diameter 3.2 cm and rotating at a speed of 10 rpm. Trained rodents which stayed on a rod for one minute were injected with the prepared compounds (**1-14**) by *i.p.* route at a dose of 300 mg/kg. Motor impairment was indicated by the inability of the animal to maintain equilibrium on the rod for at least 1 min in each of the three trials.

*Statistical analysis*

Data for anti-convulsant activity are expressed as mean ± SEM. One-way ANOVA followed by Dunnett’s test were used to test the statistical differences between vehicle control and treatment groups. A probability value of less than 0.01 was considered as significant.

*Computational methodology*

Molegro Virtual Docker (MVD) was used to perform the docking studies in order to probe the anti-convulsant actions of synthesized sulfonamide benzothiazole derivatives (**1-14**) based on their structural features (24). The binding modes, binding affinities and orientation of the target compounds at the ion gated channel receptor was predicted with the help of docking score and number of hydrogen bonds formed with the amino acid residues. 

*Ligand preparation*

The 2D chemical structures of molecules were drawn using Marvin Sketch 5.11.0 and converted to 3D structures by optimization method. After the addition of explicit hydrogens the structures were saved as MDL Mol File (*.mol2).

*Enzyme setup and procedure*

Molegro Virtual Docker 4.0.2 program was used to perform the docking studies. All the five chains *i.e. *A, B, C, D and E of the crystal structure of Ion Channel receptor (2BG9) were selected for the docking studies. The structure was downloaded from protein data bank and docked with synthesized compounds. After docking, the compounds were ranked according to their docking scores and then visualised inside the protein’s binding pocket to obtain information about the fitting and nature of interactions with the active sites of main amino acid residues. The Hydrogen bond network was optimized in the subsequent stage of protein preparation by reorienting hydroxyl group, water molecules, and amide groups of the amino acid residues which are believed to act as gate for the ligand entrance in to the 2BG9 active sites such as Arg 277, Gln 276, Glu 262, Glu 274, Tyr 223, Lys 269 and Ser 266. 

## Results and Discussion

*Chemistry*

The synthesis of benzothiazole coupled substituted benzene sulfonamides were accomplished in two simple steps. In the first step of the synthetic Scheme 1 (step I),2-(4-aminophenyl) benzothiazole was prepared by simply triturating the reactants *viz.* 2-aminothiophenol (0.008 mM) with *para* -anthranilic acid (0.008 mM) in the presence of molecular iodine (0.008 mM) catalyst. The progress of a chemical reaction to produce the desired cyclised product *i.e.*, 2-(4-aminophenyl) benzothiazole was monitored on a TLC plate in a toluene: ethyl acetate: formic acid (5:4:1) solvent system. Upon successful completion of the reaction, the purity of the synthesized compound was further ascertained by spectral and elemental analysis data which were found to be consistent with the chemical structure of 2-(4-aminophenyl) benzothiazole. To study the effect of amino group on structure activity relationship at *meta-*position, 2-(3-aminophenyl) benzothiazole was also synthesized in the similar fashion.

The title compounds *N*-(4-(benzothiazole-2-yl) phenyl) ¾ substituted benzenesulfonamides were synthesized as per step II by condensing 2-(3/4-aminophenyl) benzothiazoles with various substituted sulfonyl chlorides in pyridine and acetic anhydride in presence of heat. The different substituted benzene sulfonyl chlorides were prepared by reacting chlorosulfonic acid (0.77 mol) and substituted benzene derivatives (0.148 mol) at standard reaction conditions. 

*Physicochemical and spectral data of synthesized compounds*

The purity of the prepared compounds was checked with the help of melting point, solubility and thin layer chromatography (TLC). To ascertain that all prepared compounds have different chemical nature than the respective parent compounds, identification and characterization of the synthesized compounds was carried out by recording the spectral data using Infrared (IR), Nuclear Magnetic resonance (NMR) and Mass spectrometry techniques. 

The IR spectra of compounds **1-14** showed absorption bands for N-H_str_ and C=N groups in the range of 3425-3318 and 1590-1658 cm^-1^ respectively. IR spikes in the region of 3119-3068 and 1389-1312 cm^-1^ were observed due to -SO­_2_NH and C-N groups respectively. The ^1^H NMR spectra of all compounds showed a broad singlet at δ 4.19-4.7 due to –NH proton. In compounds **5**, **7**, **12** and **14**, an additional singlet due to the three protons of a methyl group was observed at δ 2.38-2.67. The aromatic protons appeared as multiple peaks at δ 7-8.The ^13^C NMR spectra accounted for all the carbon atoms in the synthesized compounds. All the compounds showed signals for 18 aromatic carbons (115.1-151.2). A downfield signal around 168.6-170.1 was observed due to a carbon atom in C=N of a thiazole ring. A distinct signal was also observed for methyl carbon in compounds **5**, **7**, **12**, **14**. In mass spectra of compounds, molecular ion (M^+^) and isotopic peaks (M^+^+2) were also obtained in reasonable intensities which further confirmed the chemical structure of compounds.

*Anti-convulsant activity*

The anti-convulsant activity of the synthesized compounds was evaluated by employing MES method and PTZ test (60 mg/kg) in mice using phenytoin and diazepam standard drugs respectively. The results of anticonvulsant activity are presented in Tables 1-3. According to the results presented in Table 1, compound **9** was found to be the most potent, followed by **10** and **11** and then **2**,** 4**,** 8,** and **12** in MES model. Compound **9** exhibited better flexion (1.8 ± 0.37) and recovery in comparison to standard drug (3.6 ± 0.68 and 171.8 ± 2.92 respectively). While in Pentylenetetrazole (PTZ) test method, compound **8** was observed to be the most potent anticonvulsant agent (Table 3), followed by compounds **9** and **10**. From these results we concluded that the synthesized compounds with different substitutions on *N*-[4-(benzothiazole-2-yl) phenyl] 3- substituted benzene sulfonamide possess better anti-convulsant activity in comparison to 4-substituted derivatives. Further, *p*-Cl group on benzene sulphonamide exhibits better activity than *p*-Br or *p*-F groups. 

The results of neurotoxicity test indicated no motor impairment after 0.5 h, however, few compounds (**1**, **3**, **5** and **13**) showed neurotoxicity after 3 h.

*Docking studies*

The aim of the docking studies was to predict the binding affinity and interaction of sulfonamide bearing benzothiazole ligands with the target protein (PDB ID: 2BG9). Molegro Virtual Docker (MVD) is a user friendly docking software which is used effectively to perform the flexible docking of ligands into their site of action. It uses all the rotatable bonds of the ligands to produce a number of conformations from which the best mode could be selected. We tried to find a correlation between the observed biological experimental results and docking studies (Figures 3-6). The results presented in Table 4 reveals that the Mol dock score of standard ligand was -116.65 and it forms three interactions. The ligand showing three hydrogen bonds (as shown by blue doted lines) out of which two bonds are formed between O of SO_2_ of sulfonamide with N of residue Arg 277 having a distance of 2.60Å and 3.16 Å respectively while another found between N of NH of sulfonamide with O atom of Gln 276 with 2.90 Å distance. Compound **13** exhibits relatively comparable binding affinity with highest Mol dock score of -115.37 along with interactions more than standard *i.e.* five out of which two interactions were found between N of NO_2_ with N of Arg277 having distance of 3.29 and 3.25 Å respectively, another two interactions between N of NO_2_ with O of Arg277 having distance of 2.59 and 2.85 Å respectively, while the fifth interaction was found between O of SO_2_ with O atom of Ser266 having a distance of 3.45 Å. The compound **13** was followed by the compound **6**, a *meta *analogue of compound **13 **with same functional group -NO_2 _having second highest Mol dock score of -114.67 and three interactions between N and O atoms of NO_2_ with N atom of Arg 277 having distance 3.88 Å, 2.42 Å and 2.84 Å, respectively. The results of docking studies presented in Table 4 clearly indicate that compound **13** have best docking score and form maximum number of interactions (*i.e. *5), followed by compounds **6**, **11** and **3**. 

Incorporation of electron withdrawing groups (Cl, Br, F) at meta position in the aromatic ring also showed similar types of interactions. Thus, it can be proposed that substitution of aromatic ring at *meta* position with electron withdrawing groups increases the anti-convulsant activity of benzothiazole derivatives. Though, the most of the synthesized compounds showed good binding affinity for nicotinic acetylcholine ion gated receptors, reduced the seizures induced by PTZ and MES but their precise mechanism is yet to be established. Though, based on their anti-PTZ activity, it could be hypothesized that they mediate their anti-convulsant actions by increasing GABA neurotransmission in brain.

## Conclusion

Two series of *N*-(4-(benzothiazole-2-yl) phenyl) 3/4- substituted benzene sulfonamides were successfully synthesized and evaluated for their anticonvulsant activities. It was observed that *N*-[4-(benzothiazole-2-yl) phenyl] 3- substituted benzene sulfonamide possess better anti-convulsant activity in comparison to 4-substituted derivatives. Further, *p*-Cl group on benzene sulphonamide exhibits better activity than *p*-Br or *p*-F groups. Overall, compound **9** emerged as the most potent anticonvulsant agent in maximal electroshock (MES) model in mice. Molecular docking studies also supported the biological experimental results. Further studies are recommended to develop and optimize compound **9** as a lead candidate for the anti-convulsant therapy. Further studies to gain an insight into the precise mechanism of anti-convulsant actions of benzothiazole coupled sulfonamide derivatives are currently under progress in our lab.
